# Ocular Stem Cell Research from Basic Science to Clinical Application: A Report from Zhongshan Ophthalmic Center Ocular Stem Cell Symposium

**DOI:** 10.3390/ijms17030415

**Published:** 2016-03-22

**Authors:** Hong Ouyang, Jeffrey L. Goldberg, Shuyi Chen, Wei Li, Guo-Tong Xu, Wei Li, Kang Zhang, Robert B. Nussenblatt, Yizhi Liu, Ting Xie, Chi-Chao Chan, Donald J. Zack

**Affiliations:** 1 State Key Laboratory of Ophthalmology, Zhongshan Ophthalmic Center, Sun Yat-sen University, Guangzhou 510060, China; ouyhong3@mail.sysu.edu.cn (H.O.); chenshy23@mail.sysu.edu.cn (S.C.); yzliu62@yahoo.com (Y.L.); 2 Department of Ophthalmology, Stanford University, Palo Alto, CA 94303, USA; Jeffrey.Goldberg@Stanford.edu; 3 Unit on Retinal Neurophysiology, National Eye Institute, National Institutes of Health, Bethesda, MD 20892, USA; liwei2@nei.nih.gov; 4 Department of Ophthalmology, Tongji University, Shanghai 200092, China; gtxu@tongji.edu.cn; 5 Department of Ophthalmology, Xiamen University, Xiamen 361005, China; wei1018@xmu.edu.cn; 6 Department of Ophthalmology, University of California San Diego, San Diego, CA 92093, USA; k5zhang@ad.ucsd.edu; 7 Laboratory of Immunology, National Eye Institute, National Institutes of Health, Bethesda, MD 20892, USA; nussenblattr@nei.nih.gov; 8 Stowers Institute for Medical Research, Kansas City, MO 64110, USA; 9 Wilmer Ophthalmological Institute, Johns Hopkins University, Baltimore, MD 21231, USA

**Keywords:** stem cells, eye diseases, regenerative medicine, limbal stem cell deficiency, glaucoma, age-related macular degeneration

## Abstract

Stem cells hold promise for treating a wide variety of diseases, including degenerative disorders of the eye. The eye is an ideal organ for stem cell therapy because of its relative immunological privilege, surgical accessibility, and its being a self-contained system. The eye also has many potential target diseases amenable to stem cell-based treatment, such as corneal limbal stem cell deficiency, glaucoma, age-related macular degeneration (AMD), and retinitis pigmentosa (RP). Among them, AMD and glaucoma are the two most common diseases, affecting over 200 million people worldwide. Recent results on the clinical trial of retinal pigment epithelial (RPE) cells from human embryonic stem cells (hESCs) and induced pluripotent stem cells (iPSCs) in treating dry AMD and Stargardt’s disease in the US, Japan, England, and China have generated great excitement and hope. This marks the beginning of the ocular stem cell therapy era. The recent Zhongshan Ophthalmic Center Ocular Stem Cell Symposium discussed the potential applications of various stem cell types in stem cell-based therapies, drug discoveries and tissue engineering for treating ocular diseases.

Stem cell research has become a promising area of biology in the last few decades. In the last few years, it has been recognized that the stem cell potential of terminally differentiated cells can be reprogrammed. Systemic and local stem cell-based therapy has been applied in many diseases with encouraging results. Recently, there has been increased interest and activity in exploring stem cell-based therapies as potential approaches for the treatment for the degenerative eye diseases. Therapeutic mechanisms being explored include stem cell-based replacement of lost neurons and other ocular cells, restoration of neural circuits, and paracrine-mediated therapies in which stem cell-derived trophic factors are used as neuroprotective molecules to protect compromised endogenous retinal neurons from death and to induce the growth of new connections.

The eye has a unique structure (Figure 1) and is considered a relatively immune-privilege site, meaning transplanted cells are not as likely to be rejected as foreign compared with transplantation to other organs. Therefore, ocular stem cell therapy could, in theory, repopulate the eye with cells that have been destroyed, helping restore lost vision. However, despite the immense promise of ocular stem cell therapies, many hurdles remain, including re-evaluation of the concept of “immune privilege site” and understanding the exact fate of the stem cells after transplantation into the eye. In November 2014, ten international experts in the ocular stem cell field presented recent breakthroughs in ocular stem cell research at the Zhongshan Ophthalmic Center, Sun Yat-sen University, Guangzhou, China. The symposium reported on a variety of exciting ocular stem cell developments, from limbal stem cells for corneal disease, retinal ganglion stem cells for glaucoma, to retinal neuronal and Müller stem cells for retinal degenerative diseases, such as retinitis pigmentosa (RP) and age-related macular degeneration (AMD). Animal studies including the potential of ground squirrel induced pluripotent stem cells (iPSCs), 3D printing of cornea and retina, as well as immunology of ocular stem cell transplantation were also discussed.

The corneal epithelium is a unique non-keratinized epithelial cell in an orderly arrangement, which is crucial to the maintenance of corneal transparency [1]. It is widely accepted that the cornea is a self-renewing tissue maintained by limbal stem cells (LSCs) located at the limbus (Figure 1) [2,3]. LSC deficiency (LSCD) is a major cause of blindness worldwide [4]. In LSCD, the conjunctival epithelium migrates across the limbus, resulting in corneal opacity and vascularization, with consequent visual impairment. Kenyon *et al.* (1989) [5] were the first to report successful autologous transplantation of limbal tissue from the uninjured eye in cases of unilateral LSCD. However, this procedure carries a risk of inducing LSCD in the donor eye due to the need of large limbal biopsy, necessitating the *ex vivo* expansion of LSCs as an alternative.

Hong Ouyang, Professor of Cell Biology at Zhongshan Ophthalmic Center, discussed LSC research. In 1999, LSCs were cultured on a feeder layer of lethally irradiated mouse 3T3 cells [6]. Upon transplantation, these cultured autologous LSCs permanently restored corneal transparency of patients with ocular burns [7]. Since then, many studies have been focused on characterization and expansion of LSCs [8]. In brief, ΔNp63α and ABCG2 have proved to be key genes involved in LSC self-renewal [9]. The structural keratins, CK3 and CK12 are specific markers for differentiated corneal epithelial cells, but are supposed to be negative in LSCs [10,11]. Meanwhile, murine and human studies have shown that ATP-binding cassette subfamily B member 5 (ABCB5) not only identifies mammalian LSCs but also is required for corneal development and repair [12]. Prospectively isolated human or murine ABCB5-positive LSCs possess the exclusive capacity to fully restore the cornea upon grafting to LSCD mice in xenogeneic or syngeneic transplantation models. However, no definitive marker has been identified for LSCs after decades of study.

Maintenance of tissue homeostasis relies on accurate self-renewal and lineage commitment of adult stem cells. Pathological changes in LSCs usually cause the corneal surface to turn into an opaque keratinized skin-like epithelium. It has been reported that DKK2, an antagonist of canonical Wnt signaling and Notch1, can support the maintenance of corneal epithelial cell (CEC) fate during repair in injured mouse corneas [13]. In 2008, down-regulation of the paired box protein (PAX6) associated with the abnormal epidermal differentiation found in human corneal diseases such as Stevens-Johnson syndrome, chemical burns, and aniridia was reported [14]. Recently, by using a novel feeder-cell-free LSC expansion protocol, Ouyang and her colleagues suggested a central role of the Wnt7A-PAX6 axis in CEC fate control. Moreover, exogenous expression of PAX6 in human epidermal cells is sufficient to convert them to LSC-like cells, and upon transplantation onto eyes in a rabbit corneal injury model, these reprogrammed cells are able to replenish CECs and repair damaged corneal surface [15]. In summary, as a pleiotropic transcription factor guiding eye morphogenesis, PAX6, together with Notch, Wnt, and TGF-β signaling pathways, are essential in determination of the corneal epithelial phenotype [6].

Ouyang suggested that stem cell-based therapy offers promising benefits to corneal regenerative medicine. In particular, LSCs have been approved as a commercial product for eye burns in Europe. In addition, in an effort to treat bilateral LSCD, a tissue-engineered cell sheet composed of autologous oral mucosal epithelium has been applied as a substitutive source of cells for the reconstruction of the corneal surface [16,17,18]. iPSCs have also been cultured and differentiated into cells with substantially elevated expression of several putative LSCs markers, including ABCG2 and ΔNp63α [19,20]. However, researchers have not yet tested the potential of iPSCs in treating ocular surface diseases in patients or in experimental animals.

Wei Li, Professor of Ophthalmology at the Eye Institute of Xiamen University, discussed the corneal stem cell niche that is located in a defined limbal structure termed the Palisades of Vogt [21]. This niche could regulate LSC self-renewal and fate decision. Ex vivo expansion and transplantation of tissue engineered corneal epithelium has been utilized in LSCD. In order to achieve efficient expansion and normal differentiation of LSCs, Li and his group have been working to fabricate a surrogate niche in terms of oxygen tension, liquid level, carrier matrix, as well as stromal cells. Oxygen concentration has been shown to be crucial in the proliferation and differentiation of various types of stem cells. Li and his group have found that corneal limbal epithelium cultured at the air-liquid interface under normoxic conditions can induce hyperproliferation and abnormal epidermal differentiation, *i.e.*, squamous metaplasia [22]. They have further reported that air exposure under hypoxic conditions can increase proliferation and inhibit the abnormal epidermal differentiation of corneal limbal epithelial cells through activation of the Notch signaling pathway and inhibition of the p38 MAPK signaling pathway. Li and his group have applied hypoxia conditions coupled with air exposure to successfully generated corneal epithelial cell sheets with normal phenotype [23].

Amniotic membranes (AMs) have been broadly used as carrier for *ex vivo* LSC expansion by functioning as a surrogate niche. Previous studies used cryopreserved AMs, which contain devitalized stromal and/or epithelial cells [24]. Li and his group evaluated the efficacy of the fresh denuded AM with live stromal cells (LAM) for *ex vivo* expansion of LSCs, and found that limbal epithelial cells growing on LAM exhibited more homogeneous, compact, and smaller in size. The colony formation efficiency of the cells expanded on LAM was significantly higher than that on the dead denuded AM (DAM) lacking live stromal cells. The LAM increased long-term BrdU labeled LSCs but not short-term BrdU labeled differentiated corneal epithelial cells. K14, p63, and Ki67 expression was higher in epithelial cells expanded on the LAM. K12 was negative in the basal epithelium on the LAM, while there was strong immunoreaction in the epithelium on the DAM. Furthermore, the epithelial cells on the LAM could be easily separated as an intact cell sheet and successfully used to reconstruct the ocular surface in the LSCD rabbit model, while the cells on the DAM could not generate intact cell sheets. The data from Li’s team indicates that the LAM can facilitate *ex vivo* LSC expansion possibly via a niche mechanism. Currently they are utilizing the culture system combining air-exposure, low oxygen, and the LAM culture system to generate tissue engineered corneal epithelium for a clinical trial, which shows promising outcome in the treatment of LSCD. Jeffrey L Goldberg, Professor and Chair of Ophthalmology at Stanford University, presented an update on the progress in using stem cells for glaucoma treatment. Retinal ganglion cells (RGCs, Figure 2) degenerate in glaucoma and other optic neuropathies, and are not replaced in humans or other adult mammals. Cell therapies are being explored for two components of glaucoma [25,26,27]. First, trabecular meshwork cells either expanded from primary tissues or derived from stem cell populations, are being studied for repopulating the collagen beams in the trabecular meshwork. Refreshing the cellular content in the trabecular meshwork is currently hypothesized by many to be a strong and perhaps durable approach to lowering intraocular pressure [28]. Second, stem cells are being studied actively to treat the RGC degeneration in glaucoma and in other optic neuropathies [29]. Early in degenerative disease, stem cells could be used to provide neurotrophic survival and growth signals and thereby counteract the degenerative process [26]. This would provide a neuroprotective effect and slow the course of vision loss and perhaps even restore some vision. Many stem cell populations have been demonstrated to secrete a variety of neurotrophic factors that could independently or coordinately promote RGC survival and growth of retinal ganglion cells.

Late in glaucoma or other optic nerve degenerations, however, a significant number of RGCs will have already died. Neurotrophic or neuroprotection approaches will be less useful at such later stages, but cell replacement therapies will be very promising for vision restoration [25,30]. Here, the challenge is scientifically much greater: stem or progenitor cells must be coaxed to differentiate into retinal ganglion cells, and to integrate into adult retinal circuits [31].

Goldberg presented his laboratory group’s latest work on RGC transplantation and integration *in vivo* in rodent models. In collaboration with his collaborator Ken Muller at the University of Miami, and led by his graduate student Praseeda Venugopalan, his team began by asking whether RGCs can be transplanted and integrate into the mature retina [32,33]. They used a normal, uninjured rat recipient to investigate whether transplanted RGCs can integrate into the mature retina, by transplanting GFP-labeled donor RGCs *in vivo* by intravitreal injection. Goldberg presented data demonstrating that transplanted RGCs acquired morphologic features of endogenous ones, with axons growing towards the optic nerve head of the host retina and dendrites extending into their target zones in the inner plexiform layer. Preliminary data showed GFP+ axons extending within the host optic nerves and optic tract to the superior colliculus and the lateral geniculate nucleus, two primary synaptic targets in the brain. Goldberg also shared data from electrophysiological recordings from transplanted RGCs, which demonstrated their electrical excitability. Light responses were also similar to host ON, ON-OFF and OFF RGCs, although less rapid and with greater adaptation. These data presented a promising approach to developing cell replacement strategies in diseased retinas with degenerating RGCs [26].

The retina is the visual information sensing, primary processing and projecting neural tissue in the eye. Similar to other parts of the nervous system in our body, all the retinal neurons are generated during development, and the adult human retina shows little regeneration activity [34]. On the other hand, the retina is vulnerable to a variety of insults ranging from physical assaults, genetic defects to aging, resulting in the degeneration of retinal neurons. Due to the lack of regeneration activity in the human retina, retinal/photoreceptor degeneration diseases are irreversible, and lack effective therapies. However, studies in the past decade indicate that mammalian retinas still possess cells that retain regeneration potential [35,36]. Exploring regeneration potential of mammalian retinas may provide alternative donor cell sources for cell replacement therapy for retinal degeneration diseases, and may even lead to endogenous repair strategies [37].

Ting Xie, an Investigator at Stowers Institute for Medical Research, Professor of Cell Biology and Anatomy at University of Kansas, and Professor of Ophthalmology at University of Missouri School of Medicine, discussed *in vivo* approaches for regenerating photoreceptors in the degenerative diseases. There are two different ways for replenishing the lost photoreceptors in age- AMD and RP patients: transplantation of *in vitro* stem cell-derived photoreceptors and regeneration *in vivo* from existing cells. Retinal pigment epithelial (RPE) cells, Müller cells and Lgr5-positive amacrine cells could be ideal cell sources for generating photoreceptors because of their *in vivo* close proximity to endogenous photoreceptors (Figure 1 and Figure 2). In the chick embryo, RPE cells can be efficiently converted to photoreceptor-like cells in the subretinal space by ectopically expressing the neurogenic gene neurogenin1 (Ngn1) or Ngn3 [38]. Similarly, forced expression of Ngn1 or Ngn3 can also successfully transform RPE cells into photoreceptor-like cells in the mouse eye [39]. Two main challenges remain for using RPE trans-differentiation into functional photoreceptor cells: the trans-differentiated photoreceptor-like cells fail to integrate into the outer nuclear layer, and their functionality has never been directly tested *in vivo*.

Xie further emphasized the potential of Müller cell as a source of retinal stem cells (RSCs). In the adult zebrafish retina, Müller cells have been shown to have remarkable ability to produce new retinal neurons, including photoreceptors, in response to light-induced injury [40,41]. Recent studies have uncovered the major signaling pathways and factors controlling Müller cell-mediated retinal neuron regeneration, including FGF-MAPK, Wnt-β-catenin, Notch and JAK-STAT signaling pathways as well as the key transcription factor ASCL1a [42]. Similarly, Müller cells undergo activation and proliferation in the postnatal chick retina in response to injury, generating Müller cells and undifferentiated retinal progenitor-like cells [43,44]. In the adult mouse retina, Müller cells also show similar gene expression profiles to developing mitotic retinal progenitors [45]. FGF2 promotes the proliferation of Müller cells to generate retinal amacrine cells in the adult mouse retina in response to NDMA-induced injury [36]. In addition, the addition of Wnt3a and retinoic acid can also promote the generation of various retinal neurons by Müller cells in response to NMDA-induced injury [35,46]. Interestingly, forced expression of Ascl1 can promote Müller cells of young mice to generate retinal neurons, including amacrine cells, bipolar cells and photoreceptors [47]. A recent study has reported that Lgr5-positive glycinergic amacrine cells in young and old mice can generate Müller cells, bipolar cells and photoreceptors, suggesting that a subset of amacrine cells might also have the potential to regenerate retinal neurons [48]. Taken together, these findings have raised the hope that Müller cells and Lgr5-positive amacrine cells in the adult retina could potentially be activated to generate photoreceptors *in vivo*, and thereby replace the lost photoreceptor cells in AMD and RP patients. In the future, it will be important to identify critical growth factors and intrinsic factors for promoting the generation of functional photoreceptors in the degenerative adult retina.

Shuyi Chen, Principal Investigator of the State Key Laboratory of Ophthalmology, Zhongshan Ophthalmic Center, further discussed the field of endogenous retinal repair and regeneration. She pointed out that many clues to the cellular and molecular features of retinal regeneration come from studies in lower vertebrates, especially teleost fishes. The retina in fish continuously grows throughout lifetime, and can recover all types of retinal cells after injury, exhibiting tremendous regeneration ability [49]. The regeneration ability of the fish retina relies on two populations of retinal stem cells (RSCs): one population, which consists of the cells that reside in a peripheral zone to the retina called the ciliary marginal zone (CMZ) (Figure 1), is responsible for adding all kinds of retinal cells to the periphery of the growing retina; the other population consists of Müller cells in the inner nuclear layer [42,50]. Under the unperturbed homeostatic condition, Müller cells in the fish retina only occasionally proliferate to generate rod precursor cells, which migrate to the outer nuclear layer and give rise only to rods (Figure 2). Upon injury, Müller cells quickly respond by de-differentiating to a retinal progenitor cell (RPC) like state, and generate all types of retinal cells to recover the damaged retina [51,52,53]. Using versatile genetic and experimental tools developed by the zebrafish research community, a number of important intrinsic factors and extrinsic signals that govern the regeneration processes in zebrafish retinas have been discovered, which is providing interesting directions to explore to unlock the regeneration potential in the mammalian retina [54,55,56,57,58,59].

As noted above, researchers have recently focused more attention on developing Müller cells as a source of RSCs. A number of reasons support this increased interest in Müller cells: first, Müller cells in lower vertebrates function as RSCs during homeostasis and upon injury [42,50]; second, Müller cells resemble glia cells in the subventricular zone of the brain which function as neural stem cells (NSC) in the adult [60]; third, Müller cells are the last cell type generated by multipotent RPCs and they express a surprisingly significant number of molecular signatures that belong to RPCs [45,61,62]; and fourth, a few Müller cells have been found to re-enter the cell-cycle after injury [35,36]. However, unlike Müller cells in fish or NSCs in the brain, the proliferative activity of mammalian Müller cells after injury is very limited, and even more limited is their differentiation activity [35,36]. Nevertheless, Müller cells are able to actively proliferate to clonally form sphere structures and give rise to multiple types of retinal neurons, when they are released from the tissue and placed in serum and growth factors containing cell culture environment [63]. Chen’s group has established a serum free culture method that allows expansion of a population of cells from adult mouse retinas. The retinal cell lines established by this approach express typical RPC markers, actively proliferate and give rise to all types of retinal neurons and glia cells under appropriate culture conditions. When transplanted, these *in vitro* expanded retinal cells partially restore visual response to photoreceptor degenerated mice [64].

Though Müller cells share multiple molecular characteristics with retinal progenitor cells, their transcriptomes are significantly different from each other, which may explain, at least partially, the limited regeneration activity of these cells [62]. Recent advances in cell fate reprogramming studies show that forced expression of key factors can reprogram terminally differentiated somatic cells not only to pluoripotent stem cells, but also to various types of tissue-specific stem cells [65,66,67,68]. These findings suggest that certain combinations of key factors that regulate Müller cell regeneration activity in zebrafish or those factors that regulate RPC activities in mammals could be used to reprogram mammalian Müller cells to a RSC/RPC state [47,69]. Chen suggested that direct Müller cell reprogramming represents a promising new strategy to prepare donor cells for cell replacement therapy for retinal degeneration diseases, and may even make possible direct endogenous repair from a patient’s own Müller cells *in situ*.

Guo-Tong Xu, Professor of Ophthalmology and Regenerative Medicine and Dean of Tongji University School of Medicine, presented his group’s data on transplantation of (A) bone-marrow mesenchymal stem cells (MSCs) with or without gene modification; (B) adipose-derived stem cells (ADSCs); and (C) embryonic stem cells (ESCs) in rats with retinal degeneration (the RCS rats). All three donor stem-cell types, after transplantation into the subretinal space, demonstrated promising therapeutic effects in terms of protection of retinal structure and function in RCS rats. MSCs could differentiate into retinal cells and replaced some of the injured host cells. ASDCs appear to function primarily through paracrine effects. The rat ESCs (rESC) differentiated to two types of retinal progenitor cells (RPCs): the rESC-RPC1 contained more glia while the rESC-RPC2 was retinal neuron enriched [70]. The rECS-RPCs survived well in the retina of RCS rats. Xu reported that the glia enriched rESC-RPC1 obtained through early and longer adherent culture only increased the b-wave amplitude at four weeks, while longer suspension cultured cells gave rise to what appeared to be more neuronal differentiation in the rESC-RPC2 cultures which significantly improved visual function in the transplanted RCS rats [70].

Wei Li, Chief of Retinal Neurobiology Section at National Eye Institute, National Institutes of Health, presented work led by his post-doctoral fellow Jingxing Ou in which they developed a ground squirrel iPSC system to study “hibernation in a dish”. Hibernation is a trait that some animals have evolved to cope with harsh winter environments. By lowering their metabolic rate and utilizing lipid fuels accumulated during the summer and fall, mammals such as ground squirrels seal themselves underground and survive the cold winter with little food and water intake. This is an amazing biological feat, as hibernators have to alternate between two vastly different metabolic states multiple times. Studying hibernation can help us not only to understand this fascinating biological phenomenon, but also to provide invaluable information about mechanisms of metabolic regulation that are critical for adapting to metabolic challenges. Such challenges are frequently brought upon in diseases and pathological conditions.

Li’s lab studies adaptive changes in the ground squirrel retina during hibernation [71,72,73,74,75]. In many non-hibernating animals, neurons cannot cope with a body temperature substantially lower than their euthermic level. To further study the unique features of ground squirrel retinal cells and of other neurons in response to cold temperatures during hibernation, Li and his team have worked to develop a cell culture system that can easily be subjected to pharmacological and genetic manipulations. With this goal in mind, they generated several lines of ground squirrel iPSCs derived from postnatal cortical neural precursor cells and developed a system for studying intrinsic hibernating features of ground squirrel retinal cells by further differentiating these precursor cells into eyecups.

To generate iPSC lines, postnatal neural precursor cells were transduced with Sendai vectors to express the four classic Yamanaga transcription factors (POU5F1, SOX2, MYC, and KLF4; human genes). These iPSC cells were positive for expression of pluripotency markers such as OCT3/4 (POU5F1) and NANOG. Li’s team tested their pluripotency *in vitro* by differentiating them into signature cell types of the three germ layer cells (muscle cells—mesoderm, liver cells—endoderm, and neurons—ectoderm). In addition, positive results for teratoma formation further confirmed the pluripotency of the cells *in vivo*. Finally, karyotyping experiments suggested that the cell lines are genetically stable. This work by Li’s team represents the first example of successful generation of iPSC cell lines from a hibernating mammal.

Using iPSC-derived neuronal culture, preliminary data indicate that ground squirrel iPSC-derived neurons differ from human iPSC-derived neurons in that they are able to largely maintain their cytoskeletal structure when subjected to low temperature culturing (6 °C) for up to five days. Human iPSC-derived neurons, on the other hand, could not withstand low temperature culturing for as little as 10–12 h. Using pharmacology, genetics, and systems biology approaches, Li’s team is currently exploring the molecular mechanism(s) of this cold resistance feature of ground squirrel iPSC-derived neurons.

Kang Zhang, Professor of Ophthalmology and Genetics and the founding Director of the Institute for Genomic Medicine at the University of California at San Diego, summarized his work in genetics, epigenetics, stem cell and 3D printing -based therapy for retinal blindness. His group demonstrated that the HTRA1 locus at chromosome 10q26 strongly impact AMD risk [76], and showed that variations in complement factor H (CFH), HTRA1, and C3 contribute to a majority of the genetic risk for AMD and strongly predictive of advanced AMD and bilaterality [77]. Zhang also discussed the role of environmental factors, such as smoking and the influence of epigenetics in determining AMD risk [78].

Zhang believes that stem cell research shows great promise in modeling disease *in vitro* and treating ocular degenerative diseases including AMD and glaucoma [79]. Interestingly, modulation of cellular metabolism by small molecules to either enhance reprograming or inhibit pluripotent cell proliferation may have additional application in the future development of iPSC and human ESC (hESC), in particular neural precursors [80,81]. 3D printing of retina and cornea offer an exciting avenue of research and treatment [15,82]. The recent advance in genetics and stem cell therapy of ocular diseases will allow identification of high risk patients for customized intervention and treatment in the near future [83].

Donald Zack, Professor of Genetic Engineering and Molecular Ophthalmology at Wilmer Eye Institute, Johns Hopkins University presented a talk entitled “Use of Human Stem Cell-Derived Retinal Cells for Drug Discovery” in which he highlighted the power of combining human retinal stem cell systems with high content screening approaches, both for increasing our understanding of retinal development and disease pathogenesis as well as for facilitating drug discovery. Zack first described work from his lab on efforts to identify RGC neuroprotective molecules for the treatment of glaucoma and other forms of optic nerve disease (Figure 2). These initial studies, led by Derek Welsbie, made use of primary cultures of murine RGCs [84]. As a follow-up on these studies, with the goal of creating a system that might be more amenable to the study of human RGC biology, and might yield neuroprotective lead molecules more readily translatable to candidate drugs that would be efficacious and safe for use in humans, Zack described work led by Valentin Sluch on the development of an improved technology for the generation of human stem cell-derived RGCs [31]. This new approach takes advantage of CRISPR/Cas9 genome editing to generate a modified H7 embryonic stem (ES) cell line that contains a fluorescent reporter for POU4F2 (BRN3B) expression, which serves as a marker for RGC differentiation. The fluorescent reporter can be used in conjunction with fluorescence-activated cell sorting (FACS) to obtain highly purified populations of human RGC-like cells that display many of the morphological, gene expression, and electrophysiological properties of endogenous RGCs. Additionally, based on preliminary studies, these cells seem amenable for use as substrates in drug screens.

Zack also described a parallel series of studies being led by Karl Wahlin to use 3D stem cell technology to generate, via human stem cell-derived retinal cups, photoreceptor cell populations suitable for use in high content screening [85,86]. He outlined Wahlin’s success in developing improved culture conditions that allow more advanced outer segment development than previously described, and also his work in using CRISPR technology to generate lines with fluorescent reporters relevant to photoreceptor differentiation and also lines that carry retinal degeneration-associated mutations, with the goal of developing “disease-in-a-dish” models for RP and related diseases.

As a third cellular system to demonstrate the potential power of combining retinal stem cell biology with small molecule screening technology, Zack described work led by Julien Maruotti in which a high throughput quantitative polymerase chain reaction (qPCR) assay was used to identify chetomin, an inhibitor of hypoxia inducible factors, as a strong promoter of differentiation of stem cells into RPE cells [87]. This finding could have implications for developing improved methods to generate human RPE cells for use in transplantation-based clinical trials for the treatment of age-related macular degeneration (AMD) [88,89,90,91].

Lastly, Robert B. Nussenblatt, Chief of Immunology Laboratory at National Eye Institute, National Institutes of Health raised his concern on stem cell transplantation that the eye is not always “privileged”. The eye has been put forward as one of the premier areas in stem cell technology to use this approach. Of great interest has been the use of RPE to treat several disorders, including inherited retinal degenerations and age related macular degeneration. This heightened interest has often centered around the concept that the eye is sequestered from the rest of the body and entertains a “privileged” relationship with the immune system. Indeed, the eye does maintain a special relationship with the immune system, but it is rather that of constant feedback and interchange. Under normal circumstances the eye attempts to maintain a downregulatory environment and several factors in the retina provide this “privilege” [92]. Many ocular resident cells have the capacity to interact with the immune system; perhaps the one with the most possible interaction is that of the RPE. The eye under normal circumstances tries to maintain a downregulatory environment. Perhaps the best known of these is the phenomenon called ACAID, anterior chamber altered immune deviation. ACAID is a stereotypic antigen-specific systemic immune response when antigens are placed into the eye. A selective deficiency of T cell function, most often seen in delayed hypersensitivity, occurs [93]. However, this phenomenon can be easily abrogated. One important phenomenon is the expression of pro-inflammatory markers when the RPE is perturbed. Under certain circumstance such as inflammatory environment, the RPE is capable of expression of Class II antigens, as well as Toll-like receptors, Fc gamma receptors, IL-6, RANTES, MCP-1 and IL-8, the result being neutrophil and monocyte chemotactic activity [94]. These observations have been extended to disease states, including RP [95], uveitis [96], and AMD [97], where there is a significant functional change in the RPE after such perturbation or disease. Cytokine mediated activation of the RPE promotes antigen presentation instead of downregulation of the immune response [98]. Class II expression has been the hallmark of immune activation and will result in the loss immune privilege in the eye [99]. Posteriorly the placing of retinal laser burns will also abolish ocular “privilege” [100], while others have shown that unlike naïve T cells, T cells with immune memory are not controlled by ocular inhibitory environment.

Stem cell generation may present challenges as well. It has been noted that there can be abnormal gene expression in some iPSCs, so that T-cell mediated immune responses can be elicited even in syngeneic hosts [101]. A second challenge facing stem cell survival would be immunosenscence. This process is seen with aging, but in putative autoimmune diseases results in the progressive decline of the fine control usually working in the immune system. A dysfunction of both the innate and adaptive immune system is seen. Several immune alterations have been noted and these include loss of the CD28 receptor, increased interleukin-17 production, and an increase in the IL-6 receptor. All of these have been noted in patients with age related macular degeneration. An upregulation of the IL-17RC in the eye and transcripts of IL-17 and -22 have been reported in the eyes of AMD patients [102], as has a change in the ration of pro-inflammatory macrophages to those playing a regulatory role [103]. All of these changes will not only abrogate immune privilege but will create an environment in the eye that could lead to cell death. Nussenblatt stated that while stem cell transplantation does open important approaches to the treatment of disease, it also opens new challenges. It will become necessary to develop tests based on human cells and evaluations in humans, since animal models may not easily translate to the human situation. This challenge should invigorate the scientific endeavor.

The Symposium provided a welcome environment to connect among the authorities and audience. The ten leading experts shared their knowledge, perspectives, innovation and insight on ocular stem cell research. Due to the characteristics of unlimited proliferation and differentiation ability for multiple cell types, stem cells represent a promising approach for vision restoration. Different sources of stem cells used include ESCs, iPSCs, retinal progenitor cells, and LSCs. These stem cells have the potential to treat a wide range of ocular diseases, such as LSCD, glaucoma, retinal degeneration and AMD. Transplantation of stem cells in animal models of eye diseases and early clinical trials has demonstrated the effectiveness of stem-cell-based treatments. Although a successful replacement of dysfunctional cells by transplantation holds great hope for patients, problems still exist which may compromise their efficacy. For example, controlling the direction of stem cell differentiation remains a challenge, which involves the use of complex factors and takes many steps. In addition, immune rejection of grafted cells is also a considerable cause of transplantation failure.

## Figures and Tables

**Figure 1:**
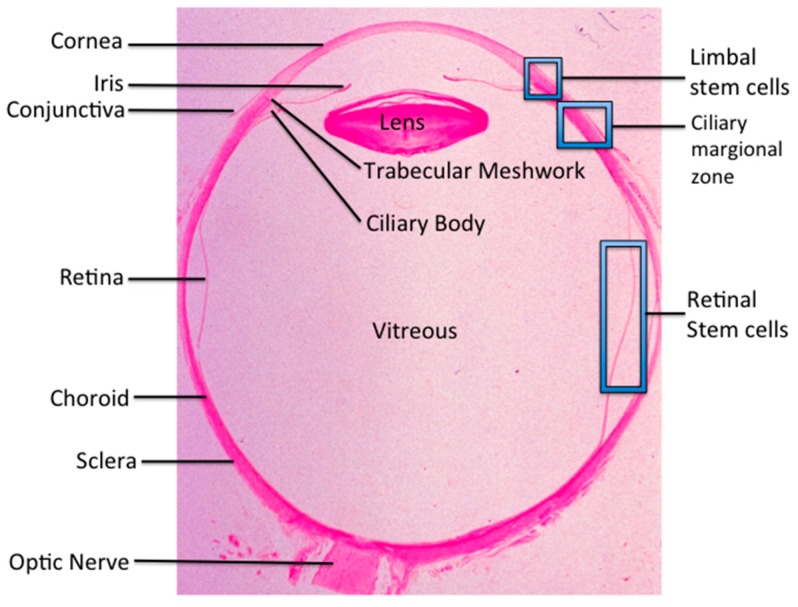
Histology of a normal eye. The square marks indicate ocular stem cell in the eye. (hematoxylin & eosin, ×25).

**Figure 2:**
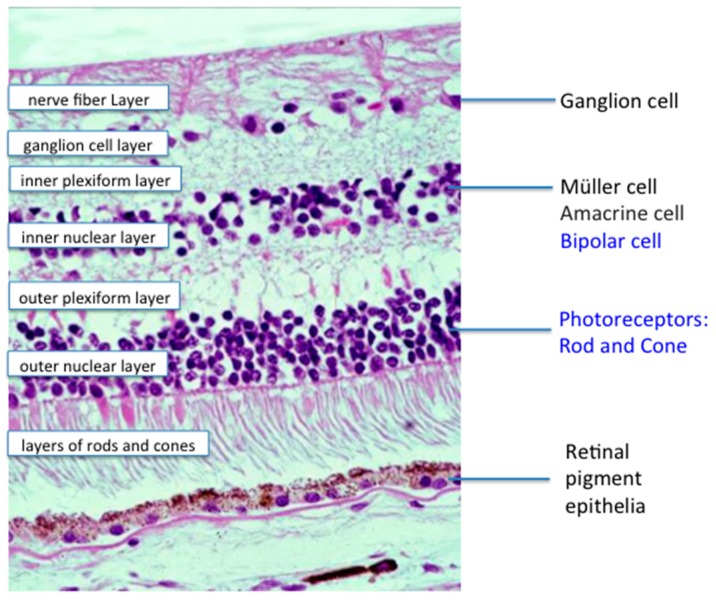
Histology of a normal retina. Retinal ganglion cell, Müller cell, and retinal pigment epithelia are the main stem cell of the retina. (hematoxylin & eosin, ×100).

## Abbreviations

AMD: age-related macular degeneration
RP: retinitis pigmentosa
RPE: retinal pigment epithelium
hESC: human embryonic stem cell
iPSC: induced pluripotent stem cell
LSC: limbal stem cell
LSCD: limbal stem cell deficiency
ABCB5: ATP-binding cassette subfamily B member 5
CEC: corneal epithelial cell
PAX6: paired box protein 6
AM: amniotic membrane
LAM: AM with live stromal cells
DAM: dead denuded AM
RGC: retinal ganglion cell
Ngn: neurogenin
RSC: retinal stem cell
CMZ: ciliary marginal zone
RPC: retinal progenitor cell
NSC: neural stem cell
MSC: mesenchymal stem cell
ADSC: adipose-derived stem cell
ESC: embryonic stem cell
CFH: complement factor H
FACS: fluorescence-activated cell sorting
qPCR: quantitative polymerase chain reaction
ACAID: anterior chamber altered immune deviation
IL: interleukin
MHC: major histocompatibility complex
